# Primary Malignant Neuroendocrine Tumour of Pleura: First Case Report

**DOI:** 10.1155/2016/5462380

**Published:** 2016-02-29

**Authors:** Anirban Das, Abhishek Pratap

**Affiliations:** ^1^Department of Pulmonary Medicine, Murshidabad Medical College, Berhampore, West Bengal 742101, India; ^2^Department of Pulmonary Medicine, Medical College, Kolkata, West Bengal 700 073, India

## Abstract

Metastatic tumours of pleura are the most common malignant tumours causing malignant pleural effusion. Lungs are the most common primary sites. Primary pleural tumours are rarely seen and diffuse malignant mesothelioma is the most common malignant tumour of pleura. Primary malignant neuroendocrine tumour of pleura is not reported in the literature. Here, we report a rare case of primary malignant neuroendocrine tumour of pleura in a fifty-two-year-old, nonsmoker female who presented with right-sided pleural effusion and ipsilateral, dull aching chest pain. Clinical presentations of inflammatory lesions like tuberculous pleuritis and benign and malignant neoplasms of pleura are indistinguishable; hence, fluid cytology, pleural biopsy, and immunohistochemistry are necessary for exact tissue diagnosis of the tumours, which is mandatory for correct treatment and prognostic assessment.

## 1. Introduction

Metastatic pleural tumours are by far the most common malignant neoplasms of the pleura and greatly outnumbering the primary pleural tumours. Lungs, breasts, and hematological malignancies are the most common primary sites for pleural metastasis. Among the primary pleural malignancies, diffuse malignant mesothelioma is the most common primary malignant tumour of the pleura. Primary pleural tumours other than mesothelioma account for less than 1% of all pleural tumours and are rarely reported in the literature, hence causing diagnostic and management dilemma [1]. In most cases, they are diagnosed as incidental during histopathological examination of pleural tissue, obtained by closed pleural biopsy or obtained under thoracoscopic visualization. Immunohistochemistry (IHC) further helps to establish the diagnosis of exact tissue type of pleural neoplasms. Besides malignant pleural mesothelioma, other primary malignancies of pleura are malignant solitary fibrous tumour, fibrosarcoma, liposarcoma, primary pleural lymphoma, synovial sarcoma, desmoplastic round cell tumours, primitive neuroectodermal tumours, blastoma, and so forth [2]. But after extensive search of literature in English, we do not find any report of primary malignant neuroendocrine tumour of pleura. Here, we report the first case of primary malignant neuroendocrine tumour of pleura in a fifty-two-year-old female, presenting with isolated right-sided pleural effusion.

## 2. Case Report

A fifty-two-year-old, nonsmoker female presented with right-sided severe dull aching chest pain, dry cough, and exertional dyspnoea for four months. Severity of chest pain was gradually increasing and disturbs her sleep at night. Shortness of breath was initially of mMRC grading 2; later it was increased to grade 3. There was no history of hemoptysis, fever, weight loss, and loss of appetite. There was no past history of pulmonary tuberculosis.

On examination, general survey revealed no abnormality. Her temperature was 97.2°F, pulse rate, 84 beats/minute, respiratory rate, 24 beats/minute, and blood pressure, 124/80 mmHg. Examination of respiratory system revealed diminished movement of chest wall on right side, tracheal shifting to left, apical impulse in left fifth intercostal space, 1.5 cm lateral to the left midclavicular line, stony dull percussion note over right chest wall, absent vesicular breath sound, and diminished vocal resonance on right side, suggestive of right-sided pleural effusion. Examination of other systems revealed no abnormality.

**Figure 1:**
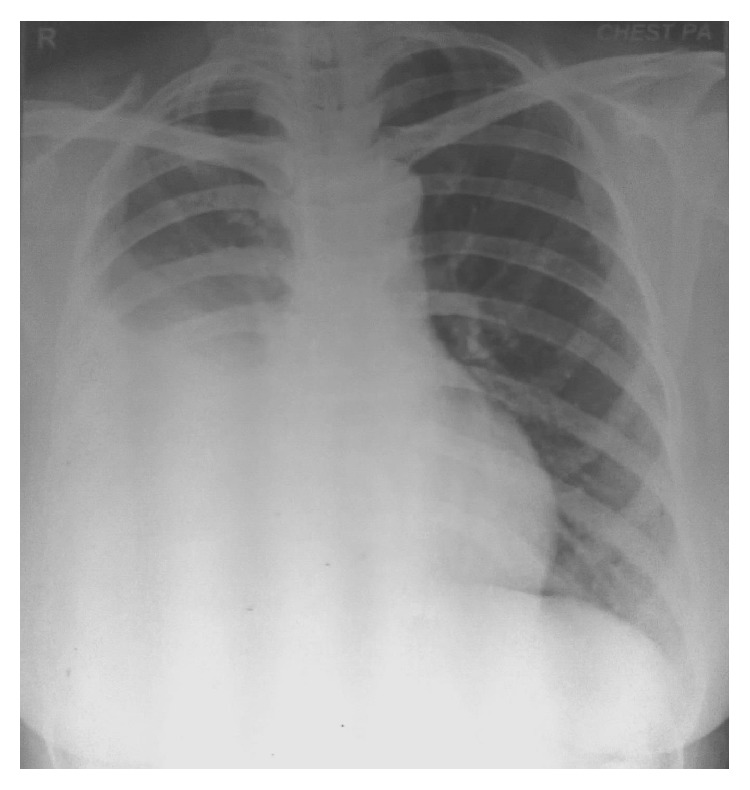
CXR, PA view showing right-sided pleural effusion.

**Figure 2:**
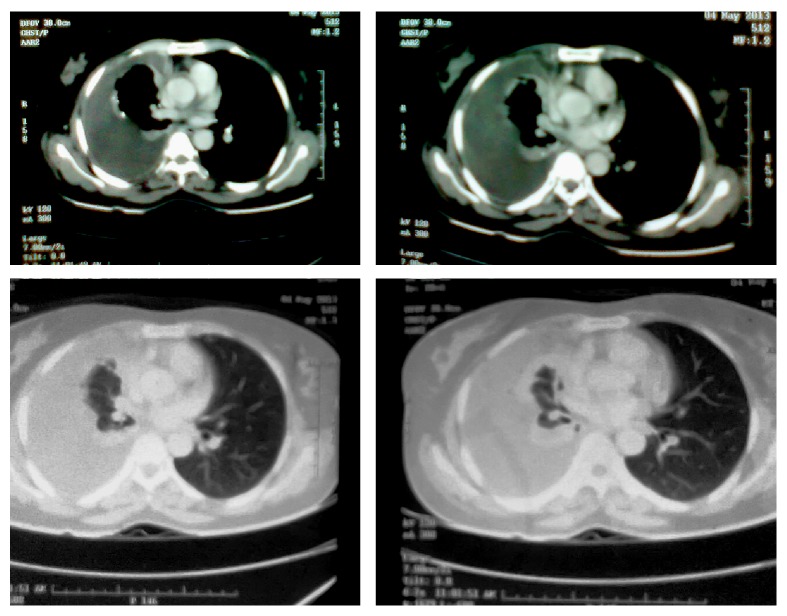
CECT of thorax showing right-sided pleural effusion.

**Figure 3:**
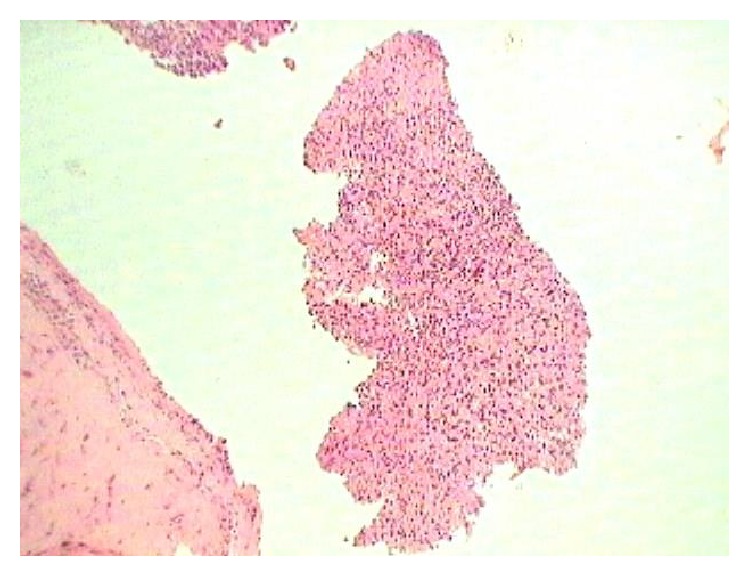
Microphotograph of histopathology of pleural biopsy tissue showing diffuse sheets of round to oval cells with hyperchromatic nuclei and scant to moderate amount of cytoplasm, focal spindle cells with hyperchromatic nuclei, and focal micropapillae formation, suggestive of a poorly differentiated malignant tumour (H&E stain, 40x).

**Figure 4:**
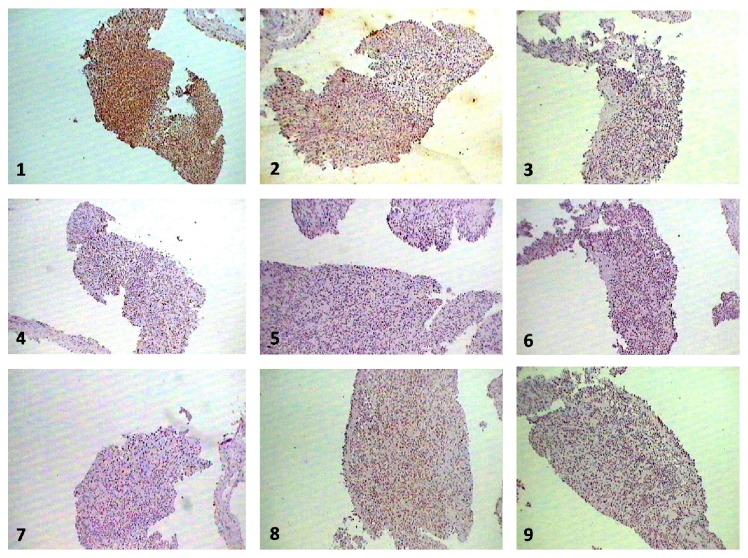
Microphotograph of immunohistochemistry showing tumour cells positive for synaptophysin (1) and occasionally for Ki-67 (2) and negative for CEA (3), CD 56 (4), calretinin (5), desmin (6), pan-cytokeratin (7), TTF1 (8), and WT1 (9) (40x).

Complete hemogram and blood biochemistry (including serotonin, gastrin, somatostatin, and glucagon) were within normal limit. Chest X-ray posteroanterior view (CXR, PA view) revealed right-sided pleural effusion (Figure 1). Ultrasound of abdomen revealed no abnormality. Pleural fluid was aspirated from right hemithorax and its analysis showed haemorrhagic, exudative, and lymphocyte predominant pleural effusion with adenosine deaminase level 16 U/L. No malignant cell or acid fast bacilli were seen in pleural fluid. Contrast enhanced computed tomography (CECT) of thorax showed only right-sided pleural effusion (Figure 2). No mass lesion or mediastinal lymphadenopathy was detected on CECT thorax. Closed pleural biopsy was done with Abram's needle and histopathological examination of biopsy tissue taken from right parietal pleura showed diffuse sheets of round to oval cells with hyperchromatic nuclei and scant to moderate amount of cytoplasm. Few scattered large cells with abundant eosinophilic cytoplasm were present. Focally spindle cells with hyperchromatic nuclei were seen. Focal micropapillae formation was also noted; features were suggestive of a poorly differentiated malignant tumour (Figure 3). Immunohistochemistry of pleural tissue showed that majority of tumour cells were positive for synaptophysin and a few cells were positive for Ki-67 (2%) and negative for pan-cytokeratin, calretinin, Wilm's tumour protein 1 (WT1), desmin, vimentin, carcinoembryonic antigen (CEA), thyroid transcription factor 1 (TTF1), and CD 56 (Figure 4). Hence, tissue diagnosis was well-differentiated malignant neuroendocrine tumour of pleura. Histopathology and IHC findings were reviewed by another pathologist of different centre, and the diagnosis was confirmed. Fibreoptic bronchoscopy (FOB) was normal. CECT of abdomen and brain revealed no abnormality. No abnormality was detected on endoscopic examination of upper gastrointestinal tract and on colonoscopy. Radionuclide bone scan did not show any metastatic deposit in bones. So, final diagnosis was primary, well-differentiated, malignant neuroendocrine tumour of pleura, presenting as isolated right-sided pleural effusion.

Six cycles of cytotoxic chemotherapy comprising of intravenous infusion of carboplatin (at an area under the concentration-time curve 6.0 on day 1, 450 mg in this case) and etoposide (100 mg/m^2^/day, 130 mg in this case on days 1, 2, and 3) were given. Along with chemotherapy, intercostal tube drainage under water seal was given in right hemithorax through fifth intercostal space at right midaxillary line, as recurrent and rapid collection of massive amount of pleural fluid occurred and repeated aspiration of pleural fluid failed to reduce the volume of pleural fluid. When there was clinicoradiological evidence of complete expansion of right lung parenchyma, chemical pleurodesis with 20 mL of 10% povidone-iodine was done. On one-year follow-up, progressive regression of pleural effusion with alleviation of chest pain was documented.

## 3. Discussion

Diffuse malignant mesothelioma is the most common primary malignant tumour of pleural tissue [3]. Metastatic carcinoma of pleura is the most common neoplastic lesions of pleura [4]. Malignant pleural tumours mainly present with pleural effusion or pleural nodules. But, it is needless to say that clinical manifestations of inflammatory lesions of pleura, benign tumours, metastases, and primary malignant tumours of pleura are indistinguishable. Hence, cytological and biochemical analysis of pleural fluid, histopathology of pleural biopsy tissue, and immunohistochemistry are essential to establish the exact etiology of pleural pathology. Presence of primary tumour in other body sites definitely indicates the metastatic pleural lesion, but primary tumours cannot always be detected.

In India, which is tuberculosis endemic zone, the most common cause of pleural effusion is tuberculosis and TB is one of the important causes of exudative, lymphocyte predominant pleural effusion. If there is evidence of rapid and recurrent collection of haemorrhagic pleural effusion, malignancy should be ruled out especially in elderly individuals, and when adenosine deaminase value is extremely low, as in our case. In these cases, CECT thorax, FOB, pleural fluid cytology, closed pleural biopsy or thoracoscopic biopsy, and immunohistochemistry are a must to confirm the diagnosis.

Lungs are the most common primary sites for metastatic pleural effusion [5]. In our case, there was no evidence of primary tumour in other body sites which were confirmed by different investigations, available in our institution. Although F-18 fluorodeoxyglucose labeled positron emission tomography (FDG-PET)-CT scan with Ga68-SSA or octreoscan is the best investigation to detect any occult primary tumour, it cannot be done in our case due to financial constraint and unavailability of the facility in our institution [6]. As lung malignancy was almost ruled out in CECT thorax and FOB, closed pleural biopsy by Abram's needle was done in our case. Closed pleural biopsy or thoracoscopic biopsy should be done in cytology negative pleural effusion, when malignancy is strongly suspected. But it is well known that diagnostic yield of closed pleural biopsy is poor in malignant pleural effusion due to predominance of malignant deposits in mediastinal and diaphragmatic surfaces of pleura which are unapproachable by Abram's needle and also due to skip lesions, characteristic of malignant effusion [7]. Hence, thoracoscopic biopsy is better as biopsy tissue is taken under direct visualization, but again it could not be done in our case due to financial constraint and unavailability of the facility in our institution.

Among the primary malignancies of the pleura, malignant solitary fibrous tumour, synovial sarcoma, fibrosarcoma, and liposarcoma are reported in the literature, beside the diffuse malignant mesothelioma. But, primary neuroendocrine tumour of the pleura is not reported till date, although there are a few reports of metastatic neuroendocrine tumours (like carcinoids) of pleura. Neuroendocrine tumours originate from hormone producing cells of neuroendocrine system and are most commonly seen in gastrointestinal tract, brain, and lungs [8, 9]. Neuroendocrine tumors of the lungs arise from Kulchitzky cells of the bronchopulmonary mucosa [10]. They comprise typical carcinoid, atypical carcinoid, large cell neuroendocrine carcinoma (LCNEC), and small cell lung cancer (SCLC) [11]. Neuroendocrine differentiation is considered if at least one of the following features is present: argyrophilia the demonstration of neuron-specific enolase or other neuropeptides like synaptophysin or chromogranin A by immunohistochemistry or the demonstration of numerous dense-core membrane-bound granules by electron microscopy [12]. The atypical carcinoids are distinguished by the presence of tumor necrosis, anaplastic large cell nuclei with numerous mitotic figures, and have a worse prognosis. Small cell carcinoma is characterized by finely granular chromatin pattern and is associated with extremely poor prognosis. Most of the small cell carcinomas share the neuroendocrine differentiation which produces different ectopic neuropeptides. In our case, histopathology of pleural biopsy tissue showed round to oval cells with hyperchromatic nuclei majority which were positive for synaptophysin and occasionally for Ki-67 but negative for pan-cytokeratin, CD56, TTF-1, desmin, vimentin, and calretinin on immunohistochemistry. Desmin, vimentin, and calretinin are characteristic immunohistochemical markers of mesenchymal tumours which were negative in our case on IHC [13]. CD56 and TTF-1 positivity are seen in small cell carcinoma and carcinoids [14, 15]. Hence, the tumour was malignant and of neuroendocrine origin (as synaptophysin positive round to oval cells) without any evidence of primary tumour in other body sites, so the diagnosis was primary malignant neuroendocrine tumour of pleura. Platinum-based cytotoxic chemotherapy comprises carboplatin or cisplatin and etoposide is useful in the treatment of neuroendocrine tumour of pleura [16]. Intercostal tube drainage and chemical pleurodesis were successful in our case and on two-year follow-up the patient was asymptomatic and pleural fibrosis was developed on affected side.
